# Implementation of noninvasive neurally adjusted ventilatory assist in pediatric acute respiratory failure: a controlled before-after quality improvement study

**DOI:** 10.1186/s44158-021-00005-8

**Published:** 2021-09-01

**Authors:** Giovanna Chidini, Daniele De Luca, Edoardo Calderini, Stefano Scalia Catenacci, Tiziana Marchesi, Thomas Langer, Cesare Gregoretti, Giorgio Conti

**Affiliations:** 1 Pediatric Intensive Care Unit, Department of Anesthesia, Intensive Care and Emergency, Fondazione IRCCS Ca’ Granda, Ospedale Maggiore Policlinico, Milan, Italy; 2 Division of Pediatrics and Neonatal Critical Care, “A.Beclere” Medical Center, South Paris University Hospitals, APHP and South Paris-Saclay University, Paris, France; 3 Department of Pathophysiology and Transplantation, University of Milan, Milan, Italy; 4 Department of Surgical, Oncological and Oral Science (Di.Chir.On.S.), Section of Anesthesia, Analgesia, Intensive Care and Emergency, Policlinico Paolo Giaccone, University of Palermo, Palermo, Italy; 5 Dept of Anesthesiology and Intensive Care Medicine, Catholic University of Rome, A. Gemelli University Hospital, Rome, Italy

**Keywords:** Acute respiratory failure, Children, Neurally adjusted ventilatory assist, Noninvasive ventilation

## Abstract

**Backgrounds:**

Pediatric noninvasive neurally adjusted ventilatory assist (NIV-NAVA) has been shown to improve patient-ventilator interaction but no data on clinical outcomes are available. Aim of this study was to compare NIV-NAVA with noninvasive pressure support (NIV-PS) in children with acute hypoxemic respiratory failure (AHRF), in a single-center before-after study. A cohort of thirty-four NIV-PS patients (before group) admitted to our PICU within the 2 years prior NAVA introduction was compared with a cohort of thirty children treated with NIV-NAVA during implementation phase (after group). The primary end-point was intubation rate between groups. Days on mechanical ventilation, number of invasive devices, nosocomial infections, PICU/hospital length of stay (LOS), and physiological parameters at 2 and 24 h after admission were considered.

**Results:**

Intubation rate was lower in the NIV-NAVA group as compared to the NIV-PS group (*p* = 0.006). Patients treated with NIV-NAVA required fewer invasive devices (*p* = 0.032) and had lower incidence of ventilator-acquired pneumonia (*p* = 0.004) and shorter PICU (*p* = 0.032) and hospital LOS (*p* = 0.013). At 2 h, NIV-NAVA compared with NIV-PS resulted in higher paO_2_:FIO_2_ (*p* = 0.017), lower paCO_2_ (*p* = 0.002), RR (*p* = 0.026), and HR (*p* = 0.009).

**Conclusions:**

Early NIV-NAVA vs NIV-PS was associated to lower intubation rate and shorter PICU and hospital LOS. Further studies are needed in order to confirm these preliminary data.

## Backgrounds

Acute hypoxemic respiratory failure (AHRF) due to viral infection is a leading cause of admission in pediatric intensive care units (PICU) [1–4].

Traditional treatment includes endotracheal intubation and mechanical ventilation. Recent experiences from pediatric studies showed that noninvasive respiratory support (NRS) has been associated with less adverse events and mortality compared to endotracheal intubation and invasive mechanical ventilation [5–9]. Nonetheless, in small children, NRS delivered as noninvasive pressure support (NIV-PS) is often associated with the presence of asynchronies due to large leaks and intrinsic characteristics of pediatric respiratory system (high respiratory rate, low tidal volume, short neural inspiratory and expiratory times, weak inspiratory effort) [8, 9].

Consequently, NIV-PS frequently results in a poor patient-ventilator interaction with reported failure rates up to 43% [10].

Neurally adjusted ventilatory assist (NAVA) is an alternative form of ventilatory support synchronous and proportional to electrical activity of the crural diaphragm (EAdi). Of note, during invasive and noninvasive NAVA, cycling is completely under neural control. Indeed, the EAdi signal results from the activation of the respiratory centers, the conduction of the electric signal through the nuclei of the phrenic nerve, the phrenic nerves and the neuromuscular junction, and finally the activation of the muscular fiber of the diaphragm. Therefore, any pathological process involving the generation and conduction of the impulse from respiratory centers to the diaphragm fibers could interfere with the generation of EAdi signal [10–13].

Recent pediatric trials showed that patient-ventilator interaction is unequivocally improved during both invasive NAVA and noninvasive NAVA compared to conventional pneumatically controlled modes [14–17]. More recently, a short-term physiological randomized controlled trial confirmed previous studies and showed that NIV-NAVA was associated with a dramatic reduction in Asynchrony Index (AI) and all type of asynchronies, particularly ineffective triggering [17]. However, the question remains whether NAVA makes a difference in terms of clinical outcomes in this population, i.e., in terms of intubation rate and PICU and hospital length of stay.

We therefore conducted this retrospective before-after study to compare the clinical efficacy of early delivered NIV-NAVA with conventional NIV-PS to treat children with AHRF.

## Methods

### Patients and data

The study was approved by the local Ethical Committee, which waived the need for informed consent, as this was a retrospective analysis and data were managed according to hospital privacy policies. This is a before-after study including all consecutive children aging less than 5 years admitted to our PICU with AHRF and treated with NIV-NAVA as first-line NRS (NIV-NAVA group) during the period occurring between January 1, 2017, and December 31, 2018.

The “before” group (NIV-PS) consisted of children with AHRF aging less than 5 years admitted to PICU between January 1, 2015, and December 31, 2016, receiving NIV-PS with PICU ventilators as a first-line NRS. Children who received NRS with turbine-driven ventilators in nonvented or vented configuration (absence of a real expiratory valve) were excluded from the analysis [18]. Our clinical protocols for indication of NRS did not change during the study period. In both periods, children admitted to our PICU were treated with NRS if they fulfilled the following clinical criteria: presence of respiratory distress (as defined by the presence of tachypnea, chest retractions, use of respiratory accessory muscles) and hypoxemia (as defined by peripheral oxygen saturation < 94% while on oxygen therapy with oxygen therapy with Venturi mask FIO_2_ 0.4). This study represents a quality improvement project and the revised Standards for Quality Improvement Reporting Excellence (SQUIRE2) guidelines were followed through the project [19].

### Noninvasive respiratory support

In the “before group,” NIV-PS was delivered by PICU ventilators equipped with specific leak compensation algorithms (Evita V500 with Y tidal volume sensor; Evita VN500 in infant configuration with Y sensor, Draeger Luebeck; Servo I Maquet (Maquet, Solna, Sweden) in infant configuration). Pressure support level was set according to clinical criteria to obtain a reduction in chest retractions and respiratory rate (RR).

Positive end-expiratory pressure (PEEP) was titrated up to 10 cmH_2_O to obtain SpO_2_ > 94% with FiO_2_ < 0.6. The inspiratory trigger was set at maximum sensitivity level not generating autotriggering. The expiratory cycling-off was adjusted by the attending physician to obtain the best patient-ventilator synchrony, according to flow/pressure/time tracings.

In the “after study,” NIV-NAVA was delivered by Servo-I Ventilators in infant mode, equipped with EAdi module (Maquet, Solna, Sweden) and provided with a leak compensation software.

Acquisition of the diaphragm electromyography (EAdi signal) was obtained using dedicated NAVA catheters positioned in lower esophagus at the crural diaphragm [15–17, 20]. To achieve the optimal ventilator assistance in NIV-NAVA, the gain was set to obtain a reduction in chest retractions and RR. PEEP level was titrated up to 10 cmH_2_O in order obtain SpO_2_> 94% with FiO_2_ < 0.6; Edi trigger was set at 0.5 μV above the resting Edi to assist respiratory effort without responding to electrical noise. Expiratory cycling-off during NIV NAVA was fixed at 70% decay in inspiratory flow. Interfaces included full-face masks of different size (Performax Respironics Murrysville, XXS, XS and S in nonvented configuration).

According to our PICU protocols, patients were switched to invasive mechanical ventilation in presence of at least one of the following criteria: severe hemodynamic instability requiring volume load and/or inotropes, severe hypoxemic respiratory failure (failure to maintain paO_2_:FiO_2_ > 150 with FiO_2_ < 0.6), severe hypercapnia (paCO_2_ > 55 torr), and recurrent apneas.

Sedation was provided according to our PICU protocol with dexmedetomidine 0.5–1mcg/kg/h and to clinical judgment [21]. The routine medical management did not vary in the study period and included standard medical therapy.

### Data collection and definitions

Basic clinical data were extracted from electronic clinical chart (Digistat; United Medical Software, Cerbaia, Italy) in which data were taken from ventilator (tidal volume, PEEP, FIO_2_, peak inspiratory pressure), from patients monitoring system (systolic blood pressure (SBP), heart rate (HR), peripheral oxygen saturation (SpO_2_)_,_ and respiratory rate (RR)) and blood gas values. The following variables were collected at admission: PIM2, sex, age, weight, AHRF trigger, and comorbidities. Data were analyzed to confirm that clinical severity at PICU admission did not change overtime.

Days on NRS, days on invasive MV, number of invasive devices per patient (central venous catheter, arterial catheter, chest tube), severe adverse events (cardiac arrest, pneumothorax, central line associated bloodstream infection, ventilator-associated pneumonia), PICU and hospital outcome, and survival at 2 and 6 months were also recorded.

Physiological parameters (RR, SpO_2_, HR, and SBP) were collected from clinical data chart at admission, at 2 h after NRS, and at 24 h after NRS in both groups. Available blood gases values were collected at admission before NRS during oxygen therapy, early after NRS institution, and at 24 h. According to our PICU respiratory failure protocol, the evaluation of NRS success or failure encompasses clinical evaluation (reduction in RR and HR, reduction in chest retractions), improvement in oxygenation, and/or reduction in arterial carbon dioxide tension (evaluated by an arterial blood gas performed approximately 2 h after NRS start). Data were anonimously recorded and stored in a password secured spreadsheet according to the current local regulations.

### End points and definitions

The primary end point in both groups was intubation rate. Secondary end point was days on mechanical ventilation (MV), number of invasive devices (central venous catheter, arterial catheter, chest tube, bladder catheter), nosocomial infections, PICU and hospital length of stay, and survival at 2–6 months.

Ventilator-associated pneumonia (VAP) and central line-associated blood stream infections (CLABSI) were defined according to CDC 2017 Definitions [22].

### Statistics

Being a study on a new ventilator technique, no a priori sample size calculation could have been performed. Thus, we decided to enroll all patients treated with NIV-NAVA from January 2017 onwards (application period) after the completion of the physiological RCT published by our group in 2016 (implementation period). Data distribution were tested with Shapiro-Wilk analysis and analyzed with parametric or non-parametric statistics, accordingly.

Primary end point, i.e., the intubation rate between groups, was analyzed with Fisher’s exact test and Kaplan-Meier log-rank analysis. Survival at 2–6 months was also analyzed similarly, while other secondary measures were analyzed with non-parametric ANOVA or Mann-Whitney *U*-test as indicated. A *p* value < 0.05 was considered significant. Statistics were performed with SPSS 15.0 (SPSS inc, Chicago, IL, USA).

## Results

Between January 1, 2015, and December 31, 2016, 145 children aging less than 5 years admitted with AHRF received NIV-PS as a first-line NRS according to clinical criteria. In this pool, 34 received NIV-PS with PICU ventilators and were included in the study. Other children received NRS via turbine-driven ventilators or variable flow-pressure generators and were excluded from the analysis because of the differences in circuit configuration. Thirty children treated with NIV-NAVA between January 1, 2017, and December 31, 2018, were included in the treatment group. Demographic characteristics between groups in the two periods were similar confirming the lack of change in local epidemiology (Table 1).

**Table 1:** Baseline characteristics of patients

	NIV-PS *n* = 34	NIV-NAVA *n* = 30	*P value*
Patients characteristics			
Male, *n* (%)	15 (45)	15 (30)	0.768
Age, mos	12, 6–21	11, 5–22	0.181
Weight, Kg	8, 6–12	8, 7–11	0.784
PIM2	1, 1–2	1, 1–1.5	0.569
paO_2_:FiO_2_	140, 116–180	143, 119–171	0.805
paCO_2_, mmHg	46, 40–52	45, 43–54	0.724
RR, breaths min^−1^	60, 55–66	60, 33–70	0.076
HR, beats min^−1^	134, 121–143	132, 120–133	0.079
MAP, mmHg	55, 50–56	55, 55–67	0.745
Causes of AHRF			
*Viral infection, n (%)*	15 (50)	17 (56)	0.694
*Bacterial infection, n (%)*	5 (17)	3 (10)	0.694
Comorbidities			
*Mechanical ventilation at birth, n (%)*	5 (17)	3 (10)	1
*Epilepsy, n (%)*	2 (7)	2 (7)	1
*Uncorrected congenital heart disease, n (%)*	1 (4)	2 (7)	1

*NIV-PS* noninvasive pressure support, *NIV-NAVA* noninvasive neurally adjusted ventilatory assist, *PIM2* Pediatric Index Mortality 2, *paO*_*2*_*:FiO*_*2*_ arterial oxygen tension to inspired oxygen fraction ratio, *paCO*_*2*_ arterial carbon dioxide tension, *RR* respiratory rate, *HR* heart rate, *MAP* mean arterial pressure, *AHRF* acute hypoxemic respiratory failure. Data are expressed as median [1–3 IQR]

**Fig. 1:**
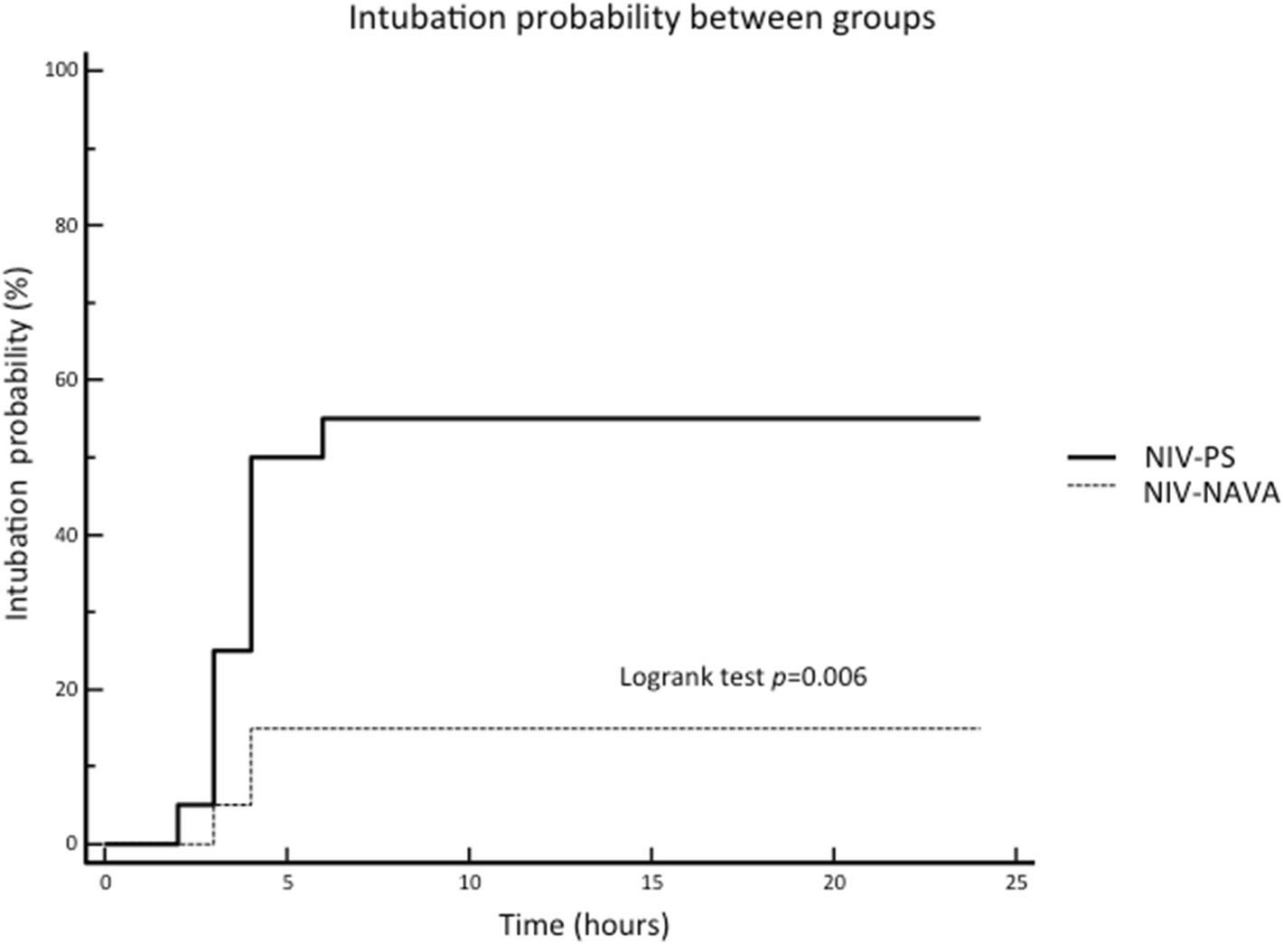
Kaplan-Meier plot of intubation probability between groups in the first 24 h. NIV-PS, noninvasive pressure support; NIV-NAVA noninvasive neurally adjusted ventilatory assist

No differences were found between the two groups in age, weight, PIM2, physiological parameters, AHRF trigger, and comorbidity. The primary end-point (i.e., the intubation rate) was significantly lower in NIV-NAVA as compared to NIV-PS (4 out of 30 [13%] vs 16 out of 34 [47%], *p* = 0.006) and as reported in Fig. 1. Outcome measures are summarized in Table 2. F_I_O_2_ and PEEP did not differ between the two cohorts (*p* = 0.084 and *p* = 0.070, respectively).

**Table 2:** Outcome variables

Variables	NIV-PS *n* = 34	NIV-NAVA *n* = 30	*P* value
FiO_2_	0.5, 0.4–0.55	0.5, 0.5–0.6	0.084
PEEP, cmH_2_O	7, 6–8	7.5 (7–8)	0.070
Peak airway pressure, cmH_2_O	16, 13–18	13, 12–14	0.003
Gain, mV	na	0.8, 0.7–1.2	
Pressurization time, ms	100, fixed	na	
Inspiratory trigger	5	0.5 mV	
Expiratory cycling, %	45, (35–55)	70% of Edi peak decay	
Tidal volume, ml/Kg/PBW	9, 8–9.5	8, 7–11	0.065
Intubation rate, *n* (%)	16(47)	4(13)	0.006
Causes to NRS failure			
*Persistent Hypoxia, n (%)*	14 (45)	4 (13)	0.040
*Intolerance, n (%)*	2 (5)	0 (0)	1
Days on invasive ventilation, *n*	7 (4–10)	3 (3–4)	0.001
VAP, *n* (%)	5 (20)	0 (0)	0.004
CLABSI, *n* (%)	1 (5)	0 (0)	1
Devices per pts, *n*	2 (0.75–4)	1 (0–2)	0.032
PICU LOS, days	8.5 (6–9.4)	5 (4–7)	0.002
Hospital LOS, days	12.3 (10–17)	8.5 (7–12)	0.013
PICU mortality, *n* (%)	0 (0)	0 (0)	1
Hospital mortality, *n* (%)	0 (0)	0 (0)	1
Two months mortality, *n* (%)	0 (0)	0 (0)	1
Six months mortality, *n* (%)	0 (0)	0 (0)	1

*NIV-PS* noninvasive pressure support, *NIV-NAVA* noninvasive neurally adjusted ventilatory assist, *FiO*_*2*_ inspired oxygen fraction, *PEEP* positive end-expiratory pressure, *PBW* predicted body weight, *PICU* pediatric intensive care unit. Data are expressed as median [1–3 IQR]

NIV-NAVA resulted in lower peak airway pressure as compared to NIV-PS (13 [12–14] vs 16 [13–17, 20] cmH_2_O; *p* = 0.003) with no differences in expiratory tidal volume. The main causes of NRS failure was hypoxia in both groups (14 [47%] vs 4 [13%], *p* = 0.040 for NIV-PS vs NIV-NAVA, respectively). The number of days on invasive mechanical ventilation was higher in NIV-PS compared to the NIV-NAVA group (7 [4–10] vs 3 [3, 4], *p* = 0.001). VAP rate and number of invasive devices per patients were lower in the NIV-NAVA group compared to the NIV-PS group (0 [0], vs. 5 [20%], *p* = 0.004, and 1 [0–2] vs 2[0.75-4], *p =* 0.032, respectively). PICU and hospital length of stay were found to be significantly shorter in the NIV-NAVA group compared to the NIV-PS group (5 [4–7] vs 9 [6–9.4] days, *p* = 0.002, and 8.5 [7–12] vs 12 [11–15] days, *p* = 0.013 respectively). No patient died neither during hospital stay, nor in the first 6 months after discharge.

**Fig. 2:**
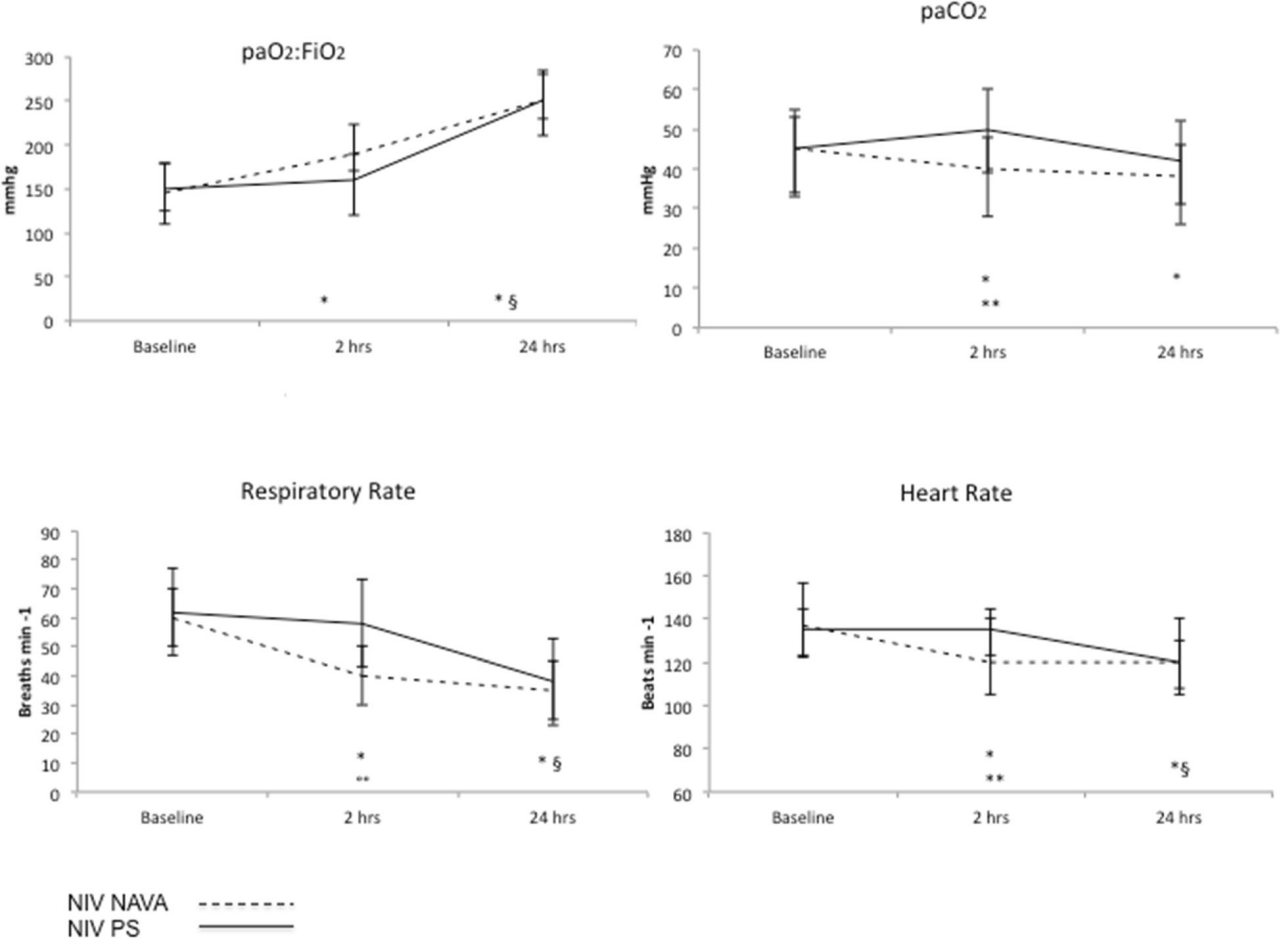
Physiologic parameters. NIV-PS, noninvasive pressure support; NIV-NAVA, noninvasive neurally adjusted ventilatory assist; paO_2_:FiO_2_, arterial oxygen tension to inspired oxygen fraction ratio; paCO_2_, arterial carbon dioxide partial pressure; RR, respiratory rate; HR, heart rate. Data are collected at baseline at PICU admission, 2 h after starting NRS in each group (*n* 30 patients in NIV-NAVA and 34 patients in NIV-PS group), and at 24 h in nonintubated patients in both groups (*n* 26 patients in NIV NAVA and *n* 14 patients in NIV-PS group). *p < 0.05: NIV NAVA comparison at 2 and 24 h vs baseline; ^§^*p* < 0.05: NIV-PS comparison at 24 h vs baseline; °°*p* < 0.05: NIV NAVA vs NIV PS at 2 h; **p < 0.001: NIV NAVA vs NIV

Physiological parameters are summarized in Fig. 2. NIV-NAVA at 2 h resulted in increased paO_2_:FiO_2_ (190 [176–250] vs 142 [115–186], *p*< 0.05), decreased paCO_2_ (40 [38–42] vs 45 [38–54] torr, *p* < 0.05), RR (40 [40–52] vs 60 [52–75] breaths min^−1^, *p* < 0.05), and HR (120 [102–120] vs 139 [124–148] beats min^−1^, *p* < 0.05) as compared to baseline. NIV-PS did not result in an early improvement in oxygenation, paCO_2_, and RR.

All physiological parameters improved at 24 h compared to baseline in nonintubated patients without differences between the 2 groups.

## Discussion

The major findings of the study are that NIV-NAVA was associated with lower intubation rate, lower number of invasive devices, shorter PICU and hospital length of stay, and an early improvement in physiological parameters compared to NIV-PS. Besides, NIV-NAVA and NIV-PS were found to be equally safe and no major adverse events due to technologic failure were reported across the entire hospital treatment.

To the best of our knowledge, this is the first pediatric study evaluating the early elective application of NIV-NAVA in a long-term treatment in comparison with NIV-PS. To date, no pediatric studies are published showing advantages of NAVA on long terms outcomes and it is not clear if a better synchronization during NRS ultimately leads to improved clinical outcomes. Data from the adult critical care population showed that a higher AI was associated with longer duration of mechanical ventilation as well as ICU and hospital mortality [23].

During the early phase of pediatric AHRF, the appropriate patient-ventilator interaction is the key point to obtain an efficient ventilatory support [16, 17, 20, 24, 25]. Previous short-term pediatric physiological studies reported a poor interaction during NIV-PS showing an AI up to 45%, a value largely exceeding the 10% cut-off currently defining severe patient-ventilator asynchrony [16, 17, 20]. In these studies, the more frequent asynchronies were ineffective triggering, premature/late cycling, and autotriggering. High AI may be due to the difficult interaction between the intrinsic characteristics of pediatric breathing pattern (low tidal volume, weak inspiratory effort, high respiratory rate, and short neural timing) and the technical performances of PICU ventilators (inspiratory trigger sensitivity, expiratory cycling setting, and leaks compensation software) [24, 25].

The technical characteristics and performances of PICU ventilators likely play a major role in determining patient-ventilator interactions. Current PICU ventilator technology, especially when large leaks are present, is often unable to detect the small variation in inspiratory flow, to allow a fast response in terms of inspiratory and expiratory cycling and to compensate for leaks around the interface.

In a recent bench-model study comparing different PICU ventilators (neonatal with proximal flow sensor and leaks compensation software, adult ventilators with or without proximal flow sensor and leaks compensations software), Vignaux et al. reported that not all ventilators performed equally in terms of delivery of tidal volume and inspiratory and expiratory delays in the presence of leaks [26].

Due to the very short pediatric neural times, the ability of the machine to optimize the patient-ventilator synchronization time by reducing the mechanical delays is crucial to deliver the support.

In the study by Vignaux and colleagues, only few neonatal ventilators equipped with enough sensitive inspiratory trigger technology were found to be able to maintain an inspiratory delay below 80 ms. Moreover neonatal ventilators demonstrated to cope better with leaks as compared to adults ventilators adapted to neonatal mode, both in terms of trigger sensitivity and cycling [26]. Besides, Vignaux et al. found that in ICU ventilator the higher the respiratory rate, the higher the amount of air trapping [27].

NAVA is a mode of ventilation where the patient’s own respiratory drive controls the timing and amount of support provided and it has been shown to optimize synchronization in preterms, full-term neonates, and small children even in the presence of large air leaks [13–17, 20, 28–30].

A correct position of the esophageal catheter for a reliable Edi signal is mandatory when using NAVA, as ventilator activation and cycling off are under direct control of Edi signal. Specific signal processing algorithms (double subtraction technique) are now incorporated in NAVA technology to achieve the highest signal to noise ratio. This allows to better trigger the ventilator, to compensate for electrodes-to-muscle distance, and to remove interferences generated from activity from intercostal or abdominal muscles and signals from ICU devices [30].

So far only three studies report data on NIV-NAVA delivered in AHRF children [16, 17, 20].

Vignaux et al. found that that NIV-NAVA resulted in shorter mechanical delays and lower Asynchrony Index (AI) (2.3% [0.7–5] vs 40% [28–65]) with the most frequent asynchronies during NIV-PS being ineffective efforts, autotriggering, and double triggering. Interestingly, the use of NIV-NAVA was associated with a reduction in all types of asynchronies [16]. Ducharme Crevier et al. reported 13 patients (3 days to 18 years) with heterogeneous AHRF where NIV was delivered both with PICU and turbine-driven ventilators. During NIV-NAVA, they found 8% (6 to 10) of total time spent in asynchrony compared to 27% (19 to 56) and 32% (21 to 38) in NIV-PS before and after NIV-NAVA, respectively. NIV-NAVA resulted in a reduction in inspiratory and expiratory delays, ineffective efforts, and autotriggering [20].

In a recent pediatric physiological RCT conducted in eighteen AHRF children, early delivered NIV-NAVA resulted in a significant reduction in AI. Indeed, NIV-NAVA led to a drastic reduction in ineffective efforts, improved interaction by reducing inspiratory and expiratory delays, and increased the synchronization time, thus significantly improving the neuroventilatory coupling [17].

These results suggest that early NIV-NAVA could be more efficient in delivering NRS compared to flow-cycled pressure support in the setting of moderate pediatric AHRF.

In the abovementioned physiologic studies, NIV-NAVA was associated with a reduction in mechanical delays and with a longer synchronization time, thus leading to a more efficient patient ventilator synchronization. In conclusions, similar to studies conducted in adults, all pediatric trials showed that patient-ventilator interaction is unequivocally improved during NAVA. What are missing so far are studies evaluating the long-term effects of NAVA on clinical relevant outcomes.

The current study suggests that NAVA could reduce intubation rate in the first 24 h from PICU admission compared to conventional ventilation. This result is corroborated by the concomitant improvement in physiologic parameters during NIV-NAVA. We can therefore argue that inspiratory and expiratory neural triggers associated with ventilator-delivered pressure controlled by EAdi improved patient machine synchronization during NIV-NAVA. This leads to better patient’s tolerance and more efficient support in respiratory function generating a reduced rate of tracheal intubation and improved hospital outcomes.

This study has several limitations that need to be addressed together with the interpretation of our data which should be done with caution.

First, this is a single center, retrospective study. The study design does not allow for undetected bias contributing to differences between variables. However because there was no time gap between the two groups and the duration of the study is relatively short, the cohorts were well matched for possible confounding variables. Treatments were also delivered according to protocolized PICU procedures that did not change during the study period [31, 32].

Second, biases related to worsening or improving in AHRF as well as the possible effect of sedation may be present and have to be taken into account in evaluating the change in gas exchange and breathing pattern.

Finally, NAVA technology is expensive and requires an extended learning curve at bedside, and whether an optimal synchronization may lead to a reduced intubation rate and better PICU outcomes remains to be confirmed by an adequately powered prospective randomized controlled trial.

## Conclusion

In this study, NIV-NAVA delivered as first-line NRS in pediatric moderate AHRF of infectious origin was associated with a lower intubation rate, shorter PICU and hospital length of stay, and early improvement of physiological parameters compared to NIV-PS. Further data from prospective RCTs are warranted to confirm these preliminary results.

## Abbreviations

AHRF: Acute hypoxemic respiratory failure
CLABSI: Central line-associated bloodstream infection
EAdi: Electrical diaphragm activity
FiO_2_: Inspired oxygen fraction
HR: Heart rate
LOS: Length of stay
MV: Mechanical ventilation
NAVA: Neurally adjusted ventilatory assist
NIV-NAVA: Noninvasive NAVA
NIV-PS: Noninvasive pressure support ventilation
NRS: Noninvasive respiratory support
paCO_2_: Partial arterial carbon dioxide tension
paO_2_:FiO_2_: Peripheral oxygen saturation to inspired oxygen fraction ratio
PEEP: Positive end expiratory pressure
PICU: Pediatric intensive care unit
PIM: Pediatric Index Mortality
RR: Respiratory rate
VAP: Ventilator-acquired pneumonia
